# Interchangeability and comparative effectiveness between generic and brand montelukast immediate release tablets after a single oral administration in healthy volunteers

**DOI:** 10.1186/s13601-015-0081-8

**Published:** 2015-11-11

**Authors:** Abdel Naser Zaid, Ayman Mousa, Nadia Ghazal, Rana Bustami

**Affiliations:** Pharmaceutical Chemistry and Technology, Department of Pharmacy, Faculty of Medicine and Health Sciences, An-Najah National University, Nablus, P.O.Box 7, Palestine; R&D Department Avalon Pharma (Middle East Pharmaceutical Industries Co. Ltd), Riyadh, Kingdom of Saudi Arabia; Naratech Pharma Consultancy, Amman, Jordan; Pharmaceutical Research Unit, Amman, Jordan

**Keywords:** Montelukast, Bioequivalence, Immediate release, Safety, Efficacy

## Abstract

**Background:**

Montelukast is a leukotriene receptor antagonist. The release of leukotrienes causes narrowing and constricting in the respiratory airways. Blocking the action of these leukotrienes, montelukast can be used for the prophylaxis and treatment of chronic asthma.

**Objective:**

The aim of this study was to evaluate the interchangeability and comparative effectiveness between a generic and a brand montelukast 10 mg immediate release tablets (Broncast^®^ and Singulair^®^, respectively) after a single oral dose among Arab Mediterranean volunteers.

**Methods:**

An open-label, randomized two-period crossover bioequivalence design was conducted in 31 healthy male volunteers with a 1 week washout between each study period and under fasting conditions. The plasma drug concentration was assessed by using a previously validated LC MS/MS method. The ratio between the generic and brand of geometric least squares means was reported for both generic and brand products. Moreover, an in vitro dissolution study was conducted on generic and brand tablets using three different pH media, and similarity and non-similarity factors (*f2* and *f1*) were calculated.

**Results:**

The used bioanalytical method was found to be linear within the range 6.098–365.855 ng/mL. The correlation coefficient was close to 0.999 during the course of the study validation. Statistical comparison of the main pharmacokinetic parameters showed the inexistence of any significant difference between generic and the brand. The point estimates (ratios of geometric means) were 111.939, 111.711, and 112.169 % for AUC_0–24_, AUC_0–∞_, and C_max_, respectively. The 90 % confidence intervals (CIs) were within the pre-defined limits of 80.00–125.00 % as specified by the FDA and EMA for bioequivalence studies. F2 and f1 were higher than 50 and lower than 15, respectively in all selected pH media.

**Conclusion:**

Broncast^®^ immediate release film coated tablets (10 mg/tablet) are bioequivalent to Singulair^®^ immediate release film coated tablets (10 mg/tablet), with a comparable safety and efficacy profile. This suggests that these two formulations can be clinically considered interchangeable. The dissolution study suggests that it could be used as premarketing quality control parameter in order to maintain the high quality of the produced product.

## Background

Montelukast (MT) is a photolabile powder with the following IUPAC name: (R,E)-2-(1-((1-(3-(2-(7-chloroquinolin-2-yl)vinyl)phenyl)-3-(2-(2-hydroxypropan-2-yl)phenyl)propylthio)methyl)cyclopropyl) acetic acid [1, 2]. MT is a potent; orally administered and active drug with anti-inflammatory properties that significantly improves asthmatic inflammation factors. It has a great affinity and selectivity to CysLT1 receptors over other respiratory tract receptors; such as the prostanoid; cholinergic; or β-adrenergic receptors. MT specifically inhibits the physiological action of LTC4; LTD4; LTE4 leukotrienes at CysLT1 receptors without any agonist activity [3–6]. Therefore; it is indicated for the prophylaxis and chronic treatment of asthma in adults and over 1 year-old pediatric patients; and it helps to control the symptoms of seasonal and perennial allergic rhinitis [4, 7, 8]. It has also demonstrated to be effective in the prevention of asthma caused by exercise in patients aged above 6 years [9]. Bioavailability studies showed that the presence of food in the GIT does not affect bioavailability parameters when an immediate release tablet is given with a standard meal [4, 10].

The oral bioavailability of MT when administered as a 10 mg immediate release (IR) film-coated tablet in adults was found to be 58–66 % [1]. In 2012, MT (Singulair^®^) immediate release tablets and other dosage forms including granules and chewable tablets were approved by the FDA for the treatment of asthma and allergic rhinitis. It is usually administered once daily in a dose of 10 or 5 mg per tablet or granules [11]. In the current study, the generic MT immediate release tablets were formulated in a single strength (10 mg/tablet). This product was developed to be as effective, and safe as the original brand. Accordingly, the objective of this study was to evaluate the bioequivalence of MT tablet formulations (10 mg/tablet) by comparing its pharmacokinetic parameters with the original brand. In addition, the in vivo safety of the two formulations (generic and brand) based on clinical and laboratory examinations and documentation of any adverse effect was also investigated.

## Methods

The study was a comparative, randomized, two-period, two-treatment, two-sequence, single dose, open label, crossover bioequivalence study of a generic MT 10 mg immediate release coated tablets (one tablet) (Avalon Pharma, Middle East pharmaceutical industries Co Itd, KSA) Versus Singulair^®^ 10 mg Film-Coated Tablets (one tablet) (Merck Sharp and Dohme Limited, Shotton Lane, Cramlington, Northumberland, NE23 3JU, UK) in healthy Subjects under fasting conditions given to healthy subjects under fasting conditions.

### Volunteers and clinical protocol

The study was conducted by Arab Pharmaceutical Industry Consulting Co. Ltd./Jordan in accordance with the requirements of the declarations of Helsinki [12], the current Good Clinical Practice (GPC) Guidelines [13] and the International Conference Harmonization (ICH) Guidelines [14]. The study protocol and the informed consent forms were approved by the Institutional Review Board (IRB). The volunteers were aged between 18 and 50 years, weighing between 57 and 93 kg with an average weight of 76 kg with body-mass index 18.5–30.0 kg/m^2^ inclusive, non-smokers or light smokers (smokers of not more than 10 cigarettes per day).The volunteers were subjected to a full medical and physical exam to confirm their healthy status and were not on any medication during the study period. The nature of the study was explained to the volunteers throughout a written informed consent, which was given to each one. The volunteers were instructed to abstain from taking drugs 1 month before starting the study, caffeine and alcohol-containing beverages for at least 16 h prior to each phase of the study and throughout the study period and to fast for at least 10 h before MT administration.

The study used an open-label, randomized two-period crossover design with a 9-day washout period between doses in 32 healthy subjects under fasting conditions. Thirty-two healthy adult male volunteers were recruited to participate in the study. 31 subjects completed the study and 31 subjects were evaluated for pharmacokinetic data. One volunteer was excluded by the clinical investigator due to an insufficient platelet count. The volunteers were randomly divided into two groups of 16 and 15 subjects. The first group was given the reference brand and the second group was given the test formulation with a crossover after the washout period. On the morning of the study, each volunteer gave a blood sample to serve as a blank for the drug assay. Each volunteer received an oral dose of the assigned MT formulation administered with 240 mL of water in the sitting position. During each period blood samples were collected from each volunteer for the calculation of the PK parameters at zero and up to 24 h after drug dosing. Each sample volume was 8 mL, 1 h before dosing, and 8 mL samples were withdrawn at following time points 0.50, 1.00, 1.50, 1.75, 2.00, 2.25, 2.50, 2.75, 3.00, 3.25, 3.50, 3.75, 4.00, 5.00, 6.00, 8.00, 12.00, 16.00 and 24.00 h after dosing. Blood samples were collected in tubes containing heparin, and centrifuged to separate the plasma fraction of the blood. The resulting plasma was immediately stored at −70 °C and analyzed by liquid chromatography tandem mass spectrometry (LC–MS/MS). Four hours after drug administration a standard lunch meal containing soup (no carrots), half a chicken, rice with mixed vegetables (no carrots), yogurt, a loaf of bread, salad (tomato and cucumber) was served and subjects had free access to water 1 h after drug administration.

### Chemicals and reagents

MT working standard (99.4 %) was supplied by Morepen Laboratories Ltd. (State Hemachal Pradesh, India) while the MT-d6 internal standard (97 %) was supplied by TRC (North York, Canada). HPLC-grade methanol and acetonitrile were purchased from Romil (Cambridge, UK), iso-propanol was obtained from Carbon Group (Cork, Ireland), extra pure formic acid was obtained from Scharlau (Port Adelaide, Australia), diethylether was obtained from JHD (Guangzhou, China) and HPLC grade water was supplied by Sartorius Purified Water (Goettingen, Germany). Control human plasma was harvested from donors.

### Tested brand and formulated tablets

The generic tablets, Broncast^®^ immediate release coated tablets (10 mg mg/tablet) were obtained from Avalon Pharma, (Middle East Pharmaceutical Industries Co Ltd, KSA) batch number 303111. Singulair^®^ immediate release coated tablets (10 mg/tablet) were obtained from Merck Sharp and Dohme (Northumberland, UK) and had a batch number of 322559.

### Instruments and chromatographic separations

A simple, fast, selective, accurate and precise High Performance Liquid Chromatographic method (Agilent Technologies, Santa Clara, United States) interfaced with an API 4000 tandem MS system (AB Sciex, Framingham, USA) was used in the analysis for the determination of MT in human plasma. The method was validated for its application to the analysis of MT in authentic plasma samples harvested after a single dose of MT 10 mg IR film coated tablets. To complete the present bioequivalence study, sampling lasted for 24.00 h after dosing; consequently a limit of quantification was 6.098 ng/mL. Montelukast-d6 was employed as the internal standard for MT, to perform its function of compensating for assay variability in the total analytical process. The stationary phase was ACE 5 CN (B2). The column used was an ACE 5 CN, 5 cm length and 4.6 mm internal diameter (HiChrom, Reading, UK). The mobile phase was composed of acetonitrile; 0.005 M ammonium acetate and formic acid in a ratio of 80:20:0.1 (v/v/v), respectively. The pH of the mobile phase was 4.30 ± 0.2. The injection volume was 10 µL and the flow rate was 0.80 mL/min. 100 % methanol was used as flushing Solution. The retention times for the drug and internal standard were 0.95 min. Dissolution test apparatus (Labindia, India) was used to assess the dissolution behavior and release of MT from tablets.

### Preparation of standard and working solutions

A drug solution was prepared by dissolving 0.08 g MT in methanol and the solution was taken up to 50.00 mL final volume to make a stock solution containing 1524.4 μg/mL. The stock solution was diluted in a serial dilution using same methanol to produce a final standard solution of 10.00 mL containing 76.22 μg/mL MT. The stock solution for the internal solution was prepared by dissolving 0.00108 g of Montelukast-d6 and the volume was made up to 100 mL with methanol to make up a stock solution 10.101 μg/mL. The internal standard stock solution was diluted in a serial dilution using methanol to produce a final standard solution of 10.00 mL containing 0.505 μg/mL. The working solutions for calibration, QC sample and IS were prepared. Prior to the extraction, each sample including: calibrators, QC samples and authentic samples were spiked with the IS solution (MT-d6). All the calibrators and the QC samples were spiked with IS in which 0.1 mL of plasma was spiked by 30 µl of the stock internal solution to get final concentration of IS of 119.5 ng/mL. Then 1 mL of ACN was added in order to eliminate proteins. Then the supernatant was analyzed.

### Bioanalytical validation procedure

The method was validated for its application to the analysis of MT in the biological fluids. Sampling lasted 24 h after drug administration in order to finalize the BE study.

The linearity assessment was performed using a series of nine standard plasma solutions previously spiked with MT (calibrator), were employed for constructing calibration curves covering a concentration ranging from 6.098 to 609.759 ng/mL. The accuracy and precision were determined by using a minimum of six replicates per concentration level. LOQ was determined by injecting a series of diluted solutions with known concentrations. In addition, stock solution stability in mobile phase was evaluated using two standard mixtures (along with internal standard). These mixtures were equivalent to the lower limit of quantification (LLOQ) and upper limit of quantification (ULOQ).

Short and long-term matrix based stabilities were evaluated using two MT concentrations, quality control low (QC_L_) and quality control high (QC_H_). Stability after freeze and thaw cycles was assessed using two QC samples. The QC samples were prepared to have low (QCL), medium (QC_M_) and high (QC_H_) concentrations (MT: 18.293, 304.879, and 457.319 ng/mL). Four QC samples were incorporated with each analysis run as unknown samples. The drug concentration in each QC sample was calculated from the calibration curve and was compared with the nominal concentration. The analysis run was accepted if at least 67 % of QC samples were within ±15 % of the nominal values.

### In vitro release of MT from film coated tablets

An HPLC method was used in order to quantify the amount of MT released from tablets. The used analytical parameters are summarized in Table 1.

**Table 1:** HPLC analytical parameters for MT

Mobile phase	Buffer solution: dissolve 1.36 g potassium dihydrogen phosphate in 1 l and adjust PH to 4.0 with 20 % v/v phosphoric acid Mix 1400 mL acetonitrile with 600 mL of buffer solution and filter through 0.45 µm membrane filter
Diluent	Mobile phase
HPLC	Waters system
Column	Inertsil ODS-2 (25 cm × 4.6 mm) 5 μm
Detector	355 nm
Flow rate	1.5 mL/min
Injection volume	50 µL
Column oven temperature	40 °C
Sample cooler temperature	NA
Standard solution preparation	Standard solution contains: 0.02 mg/mL of montelukast
Sample solution preparation	Standard solution contains: 0.02 mg/mL of montelukast
Run time	12 min
Filter	Nylon filter is suitable after discarding the first 3 mL PTFE filter is suitable after discarding the first 3 mL

The in vitro release of MT from the generic and brand immediate release film-coated tablets was conducted using USP Apparatus (II paddles) [15]. The tablets (12) were placed in the paddles of the dissolution apparatus (1 tablet/paddle) which contains 900 mL of dissolution media and the paddles were rotated at a speed of 50 rpm. The temperature was kept at 37 °C during the entire period of the dissolution study. Samples (5 mL) were taken at 5, 10, 15, and 30 min. Fresh dissolution medium (5 mL) was added to the paddles after each sample was taken. A standard was prepared of 0.011 mg/mL using same dissolution media. The samples (study and standard) were filtered through a 0.45 μm Millipore filter. A sample of 0.25 μL was injected into the HPLC apparatus. According to ICH guidelines and for the purpose of in vitro premarketing studies, this test was repeated in three different dissolution media, 01 N HCl, 4.5 acetate buffer and 6.8 phosphate buffer, respectively.

Similarity and non similarity factors, *f*_2_ and *f*_1_, were calculated in three different pH media. The *f*_2_ factor measures the closeness between two profiles and *f*_1_ measures difference between two profiles according to the following equations.

$$ f_{2} = 50 \cdot \log \left\{ {\left[ {1 + \frac{1}{n}\sum\limits_{t = 1}^{n} {(R_{t} - T_{t} )^{2} } } \right]^{ - 0.5} \times 100} \right\} $$

$$ f_{1} = \left\{ {{{\left[ {\sum\limits_{t = 1}^{n} {\left| {R_{t} - T_{t} } \right|} } \right]} \mathord{\left/ {\vphantom {{\left[ {\sum\limits_{t = 1}^{n} {\left| {R_{t} - T_{t} } \right|} } \right]} {\left[ {\sum\limits_{t = 1}^{n} {R_{t} } } \right]}}} \right. \kern-0pt} {\left[ {\sum\limits_{t = 1}^{n} {R_{t} } } \right]}}} \right\} \times 100 $$

where *R*_*t*_ and *T*_*t*_ are the percentages of drug dissolved at each time point for the reference and test products, respectively. An *f*_1_ value greater than 15 indicates significant dissimilarity, and an *f*_2_ value greater than 50 indicates significant similarity [16, 17].

### Pharmacokinetic and statistical analysis

MT plasma concentration for each of the subjects in each time point was reported and according to this data; MT plasma concentration vs. time curves were designed for both tablet products. The elimination rate constant (K_el_) was calculated from the slope of the semi-logarithmic plot of the terminal elimination phase of the blood concentration–time curve. The equation t_1/2_ = ln2/K_el_ was used to calculate the elimination half–life time (T_1/2_). The areas under the MT blood concentrations time curves from (AUC_0–24_) and the area to the infinity (AUC_0–∞_) were calculated by using the classical linear trapezoidal equation. Extrapolation to the infinity was calculated by dividing the last measurable MT blood concentration C_24_ by terminal rate constant k_el_. The AUC_0–∞_ was calculated according to the equation: AUC_0–∞_ = AUC_0–24_ + AUC_24–∞_. The statistical method used to determine the BE between both formulations was based on the procedure known as two one-sided test; to determine if transformed average values of PK parameters measured after administering both the test and reference MT tablets are comparable.

For the statistical analysis of data derived from this in BE study, one way analysis of variance (ANOVA) was used to assess the effect of formulations, periods, sequences and subjects on AUC_0–24_, AUC_0–∞_, and C_max_. A commercially available software package (Thermo Scientific Kinetica, version 5.1) was used for the calculations.

## Results

### Results of validation procedures

**Fig. 1:**
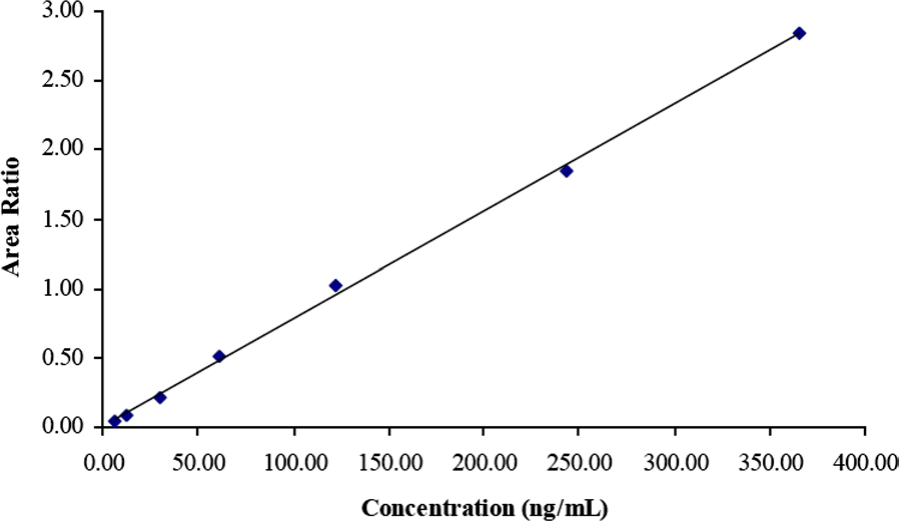
Calibration *curve* of MT

The developed and validated LC–MS/MS method was found to be effective. In fact, the relationship between concentration and peak area ratio of MT/IS was found to be linear within the range 6.098–365.855 ng/mL for MT as shown in Fig. 1. The linear equation was Y = 0.00800X − 0.00970 with a correlation coefficient of 0.998 during the course of this validation protocol. The method was sufficiently sensitive with LLOQ of 6.098 ng/mL and ULOQ of 609.759 ng/mL. The mean recovery was 109.45, 104.44 and 101.88 % for QC_L_, QC_M_ and QC_H_, respectively. The method was found to be precise and accurate for samples up to 1829.276 ng/mL. Short term stability testing (at room temperature) of MT in plasma and stock solution demonstrated that the drug was stable for up to 24 and 10 h, respectively. Freeze and thaw stability cycles showed that MT was stable after five cycles.

### Results of dissolution study and similarity factor

The release of MT from the generic and brand tablets in the recommended three pH media was within the accepted levels, since *f2* was higher than 50 and *f1* was lower than 15 as reported in Table 2.

**Table 2:** Summary of *f*_2_ and *f*_1_ for MT in dissolution media

	Dissolution medium		
	0.1 N HCl	pH 4.5 phosphate buffer	pH 6.8 phosphate buffer
*f*_2_	66	94	66
*f*_1_	11	6	14

### Results of pharmacokinetic study

**Fig. 2:**
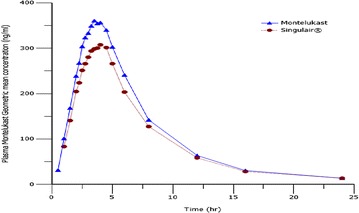
Plasma MT geometric mean concentration (ng/ml) versus time (h) *curves* and log plasma MT geometric mean concentration versus time (h) *curves* following a single oral dose 10 mg immediate release tablets

Both MT 10 mg immediate release coated tablets, Broncast^®^ and Singulair^®^, were well tolerated by all treated subjects and they were discharged in good health. Figure 2 shows the plasma concentrations of both generic and brand products indicating that the two brands are interchangeable.

A summary of the PK parameters for the two products of MT 10 mg immediate release film coated tablets. The point estimates (ratios of geometric means) were 111.939, 111.711, and 112.169 % for AUC_0–24_, AUC_0–∞_, and C_max_, respectively as reported in Table 3. The ANOVA results showed no significant statistical differences between the two formulations regarding variables such as cycles and treatment. In fact the PK parameter values lie within the EMA and FDA specified bioequivalence limits (80–125 %) [18, 19]. This study demonstrated that the test product MT 10 mg immediate release film coated tablets (one Tablet) of generic versus Singulair^®^ 10 mg immediate release film coated tablets (one Tablet) were interchangeable after single oral dose administration of each product under fasting conditions. In fact, there were no serious or significant adverse events, with both formulations being effective and sufficiently safe when administered as a single oral dose.

**Table 3:** Summary of calculated PK parameters

Parameters (unit)	Test montelukast		Reference singular®	
Efficacy results summary				
As geometric means (ranged for) C_max_ and AUC_Ratio_				
C_max_ (ng/mL)	428.910		381.900	
	176.680	783.900	80.000	792.150
AUC_0→Last_ (ng h/mL)	2731.650		2437.870	
	1386.330	6884.470	682.170	7495.260
AUC_0→inf_ (ng h/mL)	2825.270		2526.380	
	1445.510	7597.020	737.910	7963.160
As medians (ranges) for T_max_, and t_1/2_				
T_max_ (h)	3.50		3.50	
	2.00	6.00	1.50	5.00
t_1/2_ (h)	4.10		4.49	
	2.42	6.36	2.20	5.71

Parameter	Point estimate (ratio of geometric mean %)	Lower limit %	Upper limit %	CV %
Bioequivalence results summary				
C_max_	112.169	101.447	124.024	23.58660
AUC_0→Last_	111.939	101.285	123.714	23.47754
AUC_0→inf_	111.711	101.119	123.413	23.38104

## Discussion

The FDA defines a brand drug as a drug marketed under a proprietary, trademark-protected name while a generic drug is similar as the brand name drug with regard to active ingredient, dosage form, strength, route of administration, quality, safety and efficacy as assessed from the PK profile, intended use, and contains the same chemical form. However, generic versions of a drug can vary in quality of excipients and visual appearance including shape, scoring, and packaging. If all the previous criteria are met, then the two drugs are considered to be therapeutically equivalent and therefore can be safely interchangeable [18]. During the development stage of any generic oral solid dosage form, several trials and tests are carried out in order to achieve a generic product that can be as safe and effective as well as the original brand. Dissolution study of the drug in three different kinds of pH media was recommended by international guidelines in order to predict if the generic and brand could show comparable in vivo activity. In fact, these tests could be a very useful indicator as premarketing marker for each batch of oral solid drug formulation and they can be used as a biowaiver for in vivo BE studies which were assessed for the initial produced batch. In this contest, the results of dissolution showed that the generic product was comparable with the original brand. In fact, *f2* and *f1* were within the recommended criteria of biowaiver studies for MT at pH 1.2, 4.5 and 6.8 (Table 2).

Regarding the BE studies, several previous studies have tested the bioequivalence of new generic MT tablets or other dosage forms as granules and disintegrating tablets [20–23]. The PK values of our BE study were compared with similar studies that have been conducted on other populations [20, 24, 25]. It was interesting to find out that our PK values were in accordance with most of these values. However, one of these studies showed PK values lower than others as reported in Table 4 [26]. While some researchers suggest dealing with such issues by implementing personalized drug therapy, based on pharmacogenomic analysis for individual patients [27]. This would be costly and time consuming; we suggest using the PK parameters among different populations as a tool in order to adjust the recommended dose for every population accordingly. Such measures can maximize the therapeutic effects and minimize the toxic/adverse effects.

**Table 4:** Summary of PK parameters of MT (10 mg/immediate release table) from different countries

Formulation	AUC_0–24_ mean ± SD ng h/mL	AUC_0–∞_ mean ± SD ng h/mL	C_max_ ng/mL	t_1/2 h_
Generic	3251.6 ± 1221.8	3284.9 ± 1270.0	460.5 ± 170.9	3.4 ± 1.6
Brand	3162.5 ± 1537.6	3196.5 ± 1546.8	440.6 ± 227.4	3.4 ± 1.4
Generic	2443 ± 729	2530 ± 763	396 ± 120	4.9 ± 1.0
Brand	2659 ± 905	2750 ± 960	444 ± 153	4.9 ± 1.0
Generic	334.07 ± 1247.39	3.837.01 ± 1294.98	526.27 ± 178.24	3.9 ± 0.5
Brand	3733.46 ± 1248.14	3833.83 ± 1289.03	540.60 ± 190.88	4.0 ± 0.7
Generic	3707.37 ± 1 417.53	3818.73 ± 1520.90	580.40 ± 215.99	4.54 ± 0.63
Brand	3433.37 ± 1359.8	3546.19 ± 1447.11	543.13 ± 222.17	4.72 ± 0.77

The main objective of this study was to assess the bioavailability of MT 10 mg immediate release tablets produced by Avalon Pharma, versus the reference MT 10 mg (Singulair^®^ immediate release coated) produced by Sharp Merck Dohme. The two dosage forms were orally administered to 31 fasting male volunteers in order to eliminate the interference of food on drug absorption. The validated LC–MS method described above was utilized for quantification of MT 10 mg. Analytical method was successfully applied for PK studies. In fact, all validation parameters were carried out according to the ICH guidelines and they were within the accepted criteria as reported in Table 2. Regarding the efficacy of the generic product, statistical comparison of the main PK parameters, AUC_0–24_, AUC_0–∞_, C_max_ and T_max_ clearly showed the inexistence of a significant difference between test and reference tablets, in any of the assessed PK parameters. The achieved values were in accordance with the FDA and EMEA requirements for bioequivalence of generic drugs since the AUC_0–24_, AUC_0–∞,_ and C_max_ mean ratios are within the 80–125 % interval [18, 19].

Several clinical studies have highlighted the efficacy of Singulair^®^ immediate release coated tablets in the treatment of asthma in both adults and children, and for the symptomatic relief of allergic rhinitis [28–32]. Recently, Fey and et al. evaluated the efficacy and safety of montelukast 4 mg oral granules (Sandoz generic relative to Singulair^®^ mini). The extrapolated PK values were close to our results; however, the author did not specify the race of subjects that have been used in his study [33]. Accordingly, we invite authors of similar studies to specify the race of the population underlying bioavailability and BE studies in order to see if a dose adjustment could be taken into consideration for better efficacy and safety. Given the BE demonstrated for Broncast^®^ 10 mg immediate release coated tablets, this product is expected to be equally efficacious and well-tolerated. Safety is very important factor in selecting a suitable therapy, in this study the administered drugs were tolerated and all the participated volunteers completed the whole study without showing any sign of adverse effect and released in good health. Accordingly, this product offers a cheap, safe and effective treatment option for subjects with asthma or seasonal/perennial allergic rhinitis.

## Conclusion

The statistical analysis of the results which carried out on AUC_0–24_, AUC_0–∞_ and C_max_ using the ANOVA method dimonstrated that the test tablets (10 mg MT immediate release coated tablets) produced by Avalon Pharma and the reference brand tablets (Singulair^®^ 10 mg immediate release coated tablet) are bioequivalent, since they release equivalent quantities of active ingredient to the systemic circulation at equivalent rates for both AUC_0–24_ and C_max_ ratios within the 80–125 % interval proposed by FDA and the EMA agency. These results showed that the new generic tablets are clinically effective and can be safely interchanged with the original brand. Moreover, the release of MT from tablets in the selected pH media could be used as a useful premarketing quality control parameter in order to guarantee same efficacy and safety of each produced batch of this product.
